# Ultra‐widefield retinal and choroidal vascular architecture in mild cognitive impairment and normal cognition

**DOI:** 10.1002/alz.085694

**Published:** 2025-01-09

**Authors:** Suzanna Joseph, Alice Haystead, Cason B Robbins, Adam Threlfall, Tom MacGillivray, Sandra Stinnett, Dilraj S Grewal, Sharon Fekrat, iMIND research group

**Affiliations:** ^1^ Duke University School of Medicine, Durham, NC USA; ^2^ Duke Eye Center, Durham, NC USA; ^3^ Duke University, Durham, NC USA; ^4^ University of Edinburgh, Edinburgh UK; ^5^ Duke Department of Neurology, Durham, NC USA

## Abstract

**Background:**

Mild cognitive impairment (MCI) is currently a clinical diagnosis characterized by decline in memory and daily cognitive function from baseline. Exploratory studies using optical coherence tomography angiography have reported alterations in the retinal capillary plexus vessel density and attenuation of the retinal nerve fiber layer, but these results appear to be mixed. We used ultra‐widefield (UWF) imaging to evaluate retinal and choroidal vasculature and structure in individuals with mild cognitive impairment (MCI) compared to controls with normal cognition.

**Method:**

A cross‐sectional comparison of patients with MCI compared to cognitively normal controls. Scanning laser ophthalmoscopy (SLO) (California, Optos Inc, Marlborough, MA) was used to obtain UWF fundus color images. Images were analyzed with the Vasculature Assessment Platform for Images of the Retina (VAMPIRE) software. Measured metrics included vessel width gradient, vessel width intercept, large vessel choroidal vascular density, vessel tortuosity, and vessel fractal dimension.

**Result:**

131 eyes of 82 MCI patients and 231 eyes of 133 cognitively normal participants were analyzed. Both retinal artery and vein width gradients were less negative in MCI vs. controls, demonstrating decreased rates of vessel thinning towards the periphery (p < 0.001, p = 0.027). Retinal artery and vein width intercepts, a metric that extrapolates vessel width at the center of the optic disc, were smaller in MCI patients vs controls (p < 0.001, p = 0.017). The large vessel choroidal vascular density was greater in MCI patients vs controls (p = 0.025).

**Conclusion:**

When compared to controls with normal cognition, MCI patients had overall thinner retinal vasculature manifested in both the retinal arteries and veins. Furthermore, in MCI, these thinner arteries and veins attenuated at a lower rate when traveling towards the periphery, when compared to controls, perhaps because they were already more attenuated posteriorly. MCI patients also had increased choroidal vascular density.

